# The “*Fungia patella* group” (Scleractinia, Fungiidae) revisited with a description of the mini mushroom coral *Cycloseris boschmai* sp. n.

**DOI:** 10.3897/zookeys.371.6677

**Published:** 2014-01-17

**Authors:** Bert W. Hoeksema

**Affiliations:** 1 Department of Marine Zoology, Naturalis Biodiversity Center, P.O. Box 9517, 2300 RA Leiden, The Netherlands

**Keywords:** Coral reef, free-living, fungiid, solitary, budding, collection, fieldwork, Coral Triangle, Indo-Pacific

## Abstract

The recent taxonomic history of extant free-living *Cycloseris* species is briefly reviewed, resulting in the description of *Cycloseris boschmai* **sp. n.** (Scleractinia, Fungiidae) and a discussion on the validity of two other recently described species. Some *Cycloseris* species were previously considered to belong to the *Fungia patella* group, which also concerned misidentified museum specimens that actually belong to the new species. Other specimens of *C. boschmai* **sp. n.** were photographed and collected in the course of 30 years of fieldwork. The new mushroom coral is compared with other free-living *Cycloseris* species by means of an identification key. With a maximum diameter of 50 mm, it is the smallest free-living mushroom coral discovered so far. It can also be distinguished by its large primary order costae and variable colouration. Its distribution range is limited to the Coral Triangle, where it can be observed as an uncommon species on lower reef slopes.

## Introduction

Mushroom corals (Scleractinia, Fungiidae) form a common element in the fauna of most Indo-Pacific coral reefs. Depending on the species, full-grown specimens are either attached or free-living, which are character states occurring in various evolutionary lineages and therefore do not necessarily reflect phylogenetic relationships among the Fungiidae (Cairns 1984, Hoeksema 1989, 1991b, 2009, Gittenberger et al. 2011, Benzoni et al. 2012). After settlement, each fungiid individual starts as a small attached coral (anthocaulus). While corals of attached species obtain a foliaceous or encrusting growth form (Hoeksema 1989, 1993a, 2009, Gittenberger et al. 2011, Benzoni et al. 2012), those of free-living species eventually become detached from their stalk, reaching the anthocyathus stage (Wells 1966, Hoeksema 1989, Hoeksema and Yeemin 2011, Hoeksema and Waheed 2012). Owing to their charismatic appearance, abundance and large polyp size, these free-living fungiids are usually easy to find. By growing large and occurring in high densities, they may form mono- or multi-species assemblages covering extensive reef areas (Littler et al. 1997, Hoeksema 2004, 2012a, Elahi 2008, Hoeksema and Koh 2009, Hoeksema and Gittenberger 2010, Hoeksema and Matthews 2011, Hoeksema and Benzoni 2013). They can live on various kinds of reef substrates, ranging from silt to solid rock, from nearshore to offshore and from shallow reef flats to deep reef bases (e.g., Hoeksema and Moka 1989, Hoeksema 1991a, Hoeksema 2012a, 2012b, Waheed and Hoeksema 2013, in press). Mushroom corals themselves may in turn act as habitat to associated faunas consisting of molluscs, crabs, shrimps, acoel flatworms, comb jellies, and fish, some of which are host-specific (Hoeksema and Kleemann 2002, Kleemann and Hoeksema 2002, Gittenberger and Gittenberger 2005, 2011, Hoeksema and Fransen 2011, Owada and Hoeksema 2011, Bos 2012, Hoeksema et al. 2012, 2013a, 2013b, Hoeksema and Farenzena 2012, Gittenberger and Hoeksema 2013, Van der Meij and Hoeksema 2013). The various mushroom coral species vary in size (Hoeksema 1989, 1991b, Gittenberger et al. 2011) and it is obvious that corals with large surface area and thick skeletons offer most habitat space for associated fauna in contrast to small species (Hoeksema et al. 2012).

The smallest species among free-living mushroom corals appear to be among the most difficult to identify because they show relatively few distinguishing characters and much ecophenotypic variation (Hoeksema and Moka 1989, Hoeksema 1993d). Döderlein (1901, 1902) classified them as the “*patella* group” within the genus *Fungia* Lamarck, 1801. He considered them to be the most “primitive” species owing to their small size, imperforate (solid) corallum wall, and rudimentary, hardly discernible costal spines and septal dentations (see Scholtz et al. 2012). The fossil record of this *Fungia patella* group could be traced back to the Cretaceous, while its distribution ranged from eastern Africa to the west coast of America and its maximum depth was known to be over 100 m (Döderlein 1901).

Previously, species of the *Fungia patella* group were considered to belong to the genera *Cycloseris* Milne Edwards & Haime, 1849, consisting of complete corals, and *Diaseris* Milne Edwards & Haime, 1849, representing radially fragmenting corals of the same species (see Hoeksema 1989). Döderlein (1902) already considered complete and fragmenting corals to be different forms that can be found within each species. He recognized the following six species and synonyms: 1. *Fungia patella* Ellis & Solander, 1786 (including *Fungia patellaris* Lamarck, 1801, *Fungia tenuis* Dana, 1846, *Fungia hexagonalis* Milne Edwards & Haime, 1849, *Diaseris fragilis* Alcock, 1893); 2. *Fungia erosa* Döderlein, 1901; 3. *Fungia distorta* Michelin, 1842; 4. *Fungia cyclolites* Lamarck, 1816; 5. *Fungia elegans* Verrill, 1868; 6. *Fungia costulata* Ortmann, 1889. Some authors still consider *Diaseris* a separate genus (e.g. Veron 2000, Claereboudt 2006) while there is morphological evidence for their synonymy (Hoeksema 1989, Hoeksema and Waheed 2011, 2012).

Oddly, both *Fungia patella* and *Fungia patellaris* are junior synonyms of *Fungia fungites* (Linnaeus, 1756), the type species of *Fungia* (see Hoeksema 1989). However, Döderlein's (1902) photographs of his *Fungia patella* corals show that he referred to complete and fragmenting specimens of the free-living species *Cycloseris fragilis* (Alcock, 1893) and *Cycloseris sinensis* Milne Edwards & Haime, 1851 (see Hoeksema 1989). The outlines of Döderlein's (1902: pls. 1–2) smallest specimens are either circular or hexagonal.

Van der Horst (1921) mentioned six specimens of *Fungia patella* (*sensu* Döderlein), which he obtained from the Siboga Expedition collections: “The 3 *Cycloseris*-forms of Stat. 315 have both surfaces flat. Two of these specimens have the ribs obviously only at the edge of the corallum, the centrum of the under surface being irregularly covered by fine granulations. The septa of these two specimens are equal in height and very much grained. They have the appearance of *Fungia patella* var. *filigrana* Död. In the third specimen (dimensions 52 × 52 m.M.) the ribs reach the centrum, the scar of the attachment is here obvious. The edge of the corallum is slightly undulating.”

Boschma (1923a) had access to only the first two of these specimens and also to mushroom corals collected during the Danish Expedition to the Kei Islands in the Banda Sea, eastern Indonesia, and to other corals from Hawaii, among which were small specimens that according to him belonged to “Döderlein's, *patella*-group”. He recognized eight separate species besides *Fungia pattella* (*sensu* Döderlein 1902), which previously were considered synonyms: 1. *Fungia patelliformis* Boschma, 1923; 2. *Fungia fragilis* (Alcock, 1893); 3. *Fungia marginata* Boschma, 1923; 4. *Fungia distorta* (Michelin, 1842); 5. *Fungia* sp.; 6. another *Fungia* sp.; 7. *Stephanophyllia neglecta* Boschma, 1923; 8. *Fungia vaughani* Boschma, 1923. Several species were represented by complete and fragmenting shapes. Among the complete corals, several had a hexagonal outline, such as juvenile specimens of *Fungia tenuis* (Boschma 1923a: pl. 9), which actually belong to *Fungia hexagonalis* (see Boschma 1925, Hoeksema 1989). He had many specimens available to him that he identified as *Fungia marginata*; some of these were illustrated together with the original description (Boschma 1923a) and other ones in a subsequent publication (Boschma 1925). In the diagnosis of *Fungia marginata*, Boschma (1923a) refers to a circular corallum outline, although he also states that young stages are hexagonal in shape. According to him this species could be distinguished by its thick corallum margin, which might be true if compared with for instance his *Fungia patelliformis* (a synonym of *Cycloseris fragilis* (see Hoeksema 1989) but not with some other species, such as *Cycloseris costulata* (see Hoeksema 1989).

**Figure 1.**
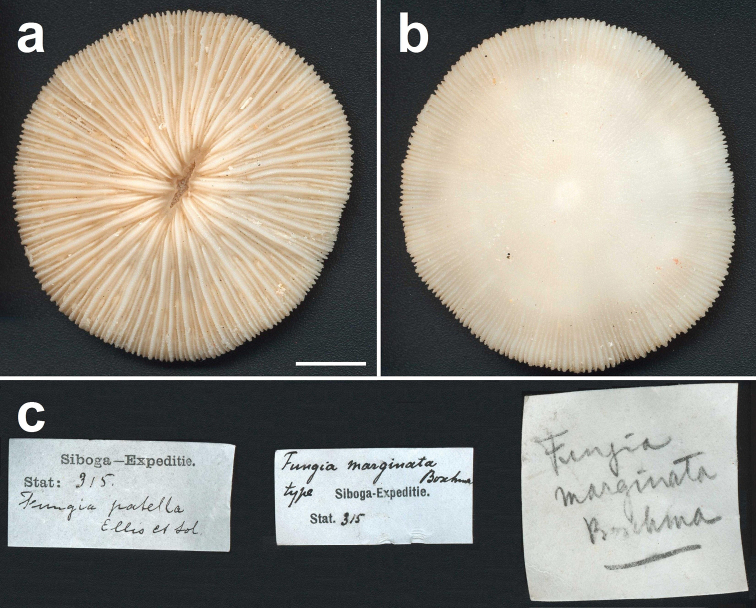
Lectotype of *Fungia marginata* Boschma, 1923 (ZMA Coel. 604, ethanol), which is a specimen of *Cycloseris costulata* Ortmann, 1889, collected at Siboga Expedition Sta. 315, Anchorage East of Sailus Besar, in the Paternoster islands, Indonesia. **a** Upper side **b** Lower side **c** Collection labels indicating the first identification by Van der Horst (*Fungia patella*) and the later one by Boschma (*Fungia marginata*). Scale bar: 0.5 cm.

**Figure 2.**
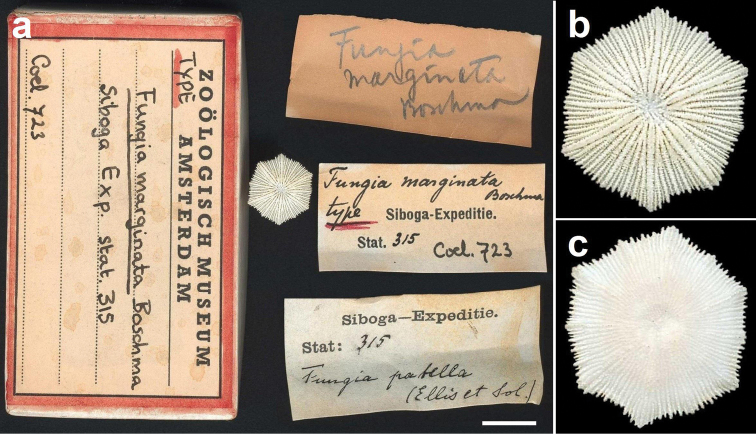
Paralectotype of *Fungia marginata* Boschma, 1923 (ZMA Coel. 723, dry), which may be a juvenile specimen of *Cycloseris costulata* Ortmann, 1889 or *Cycloseris hexagonalis* Milne Edwards & Haime, 1849, collected at Siboga Expedition Sta. 315, Anchorage East of Sailus Besar, in the Paternoster islands, Indonesia **a** Specimen with collection box and labels; scale bar: 1 cm. Collection labels indicating the first identification by Van der Horst (*Fungia patella*) and the later one by Boschma (*Fungia marginata*) **b** Close–up upper side **c** Close–up lower side.

The two specimens (ZMA Coel. collection) earlier described by Van der Horst (1921) as *Fungia patella* from Siboga Expedition Station 315 in the Paternoster Islands, Indonesia were considered syntypes (Van Soest 1979). One of these syntypes is a coral with a circular outline and a diameter of 48.5 mm (ZMA Coel. 604, herein designated lectotype; Figure 1), whereas the other is a smaller specimen with hexagonal outline, a diameter of 13 mm, and relatively thick primary costae (ZMA Coel. 723, herein designated paralectotype; Figure 2). Based on these two types, *Fungia marginata* is considered a junior synonym of *Fungia costulata* (Hoeksema 1989). Because of its doubtful identity, the juvenile specimen is not useful as type. Boschma (1923a) assumed that *Fungia costulata* does not have a solid corallum wall and has more or less equal costae and therefore described *Fungia marginata* as a species with a solid corallum wall, unequal costae, and thick corallum margin as distinguishing characters. Actually, the type of *Fungia costulata* does have a solid wall, whereas costae of all mushroom coral species may be dissimilar in size, including *Fungia costulata* (Hoeksema 1989, Gittenberger and Hoeksema 2006). Furthermore, the identity of *Fungia marginata* has never been really clear, because most subsequent authors confused it with other species (see Hoeksema 1989).

Boschma (1925) had access to many more *Fungia marginata* specimens according to the accompanying identification labels, but he did not mention them in the original species description. These specimens are still available in the collections of the Zoological Museum – University of Copenhagen (UZMK) and Naturalis Biodiversity Center (RMNH Coel.). Among these corals, several specimens are slightly larger than the syntypes and they actually do show enlarged lower order costae, although this was not clearly illustrated in his plates (Boschma 1925: pls. 5–6). These specimens are re-examined in the present study along with many small mushroom corals collected during various recent field surveys (1983–2013). The last ones were striking because of their outstanding long and thick costae and their colorful appearance in comparison with the usual brown hues found in individuals of related species (Hoeksema 1989, Gittenberger and Hoeksema 2006). Based on this unique set of characters consisting of a relatively small adult size, enlarged primary order costae and variable coloration, these corals are considered to belong to a new species, herein described as *Cycloseris boschmai* sp. n.

All species of the former *Fungia patella*-group belong to *Cycloseris*, previously considered a subgenus of *Fungia* (see Hoeksema 1989). As a consequence of recent molecular phylogenetic studies, so far 13 species have been recognized in the genus *Cycloseris*, three of which are attached (Gittenberger et al. 2011, Benzoni et al. 2012) and not part of the old *Fungia patella*-group. The new species, here described as the free-living *Cycloseris boschmai* sp. n., is the fourteenth. Specimens were either available in museum collections or were photographed and sampled during SCUBA diving in the field. In addition to a detailed description, many illustrations are presented to show the phenotypic variation of this small *Cycloseris* species, following in the foot steps of Döderlein (1902) and Boschma (1923a, 1925). For the purpose of specimen identification, a key to the presently known free-living extant *Cycloseris* species is given.

### Abbreviations

BWH = B.W. Hoeksema; Exp. = Expedition; I. = Island; Sta. = Station; MTQ = Museum of Tropical Queensland, Townsville, Australia; RMNH Coel. = Rijksmuseum van Natuurlijke Historie, Coelenterate collection (Naturalis Biodiversity Center), Leiden, the Netherlands; UZMK = Zoological Museum – University of Copenhagen, Denmark; ZMA Coel. = Zoological Museum of Amsterdam Coelenterate collection (Naturalis Biodiversity Center), Leiden, the Netherlands.

## Systematic section

### Order Scleractinia Bourne, 1900
Suborder Fungiina Verrill, 1856
Superfamily Fungiicae Dana, 1846
Family Fungiidae Dana, 1846

#### Genus Cycloseris

Milne Edwards & Haime, 1849

http://species-id.net/wiki/Cycloseris

##### Type species.

*Fungia cyclolites* Lamarck 1816. Designation by monotypy.

##### Synonymy.

*Cycloseris* Milne Edwards and Haime 1849: 72; Milne Edwards and Haime 1850: xlix; Milne Edwards and Haime 1851: 111–112; Milne Edwards 1860: 49; Tenison-Woods 1878: 328; Duncan 1883: 149–150; Quelch 1886: 119–120; Gardiner 1899: 171; Gardiner 1905: 944; Vaughan and Wells 1943: 139; Wells 1956: 388; Wells 1966: 235–236; Veron and Pichon 1980: 107–108; Ditlev 1980: 54; Nemenzo 1981: 182; Scheer and Pillai 1983: 74; Nemenzo 1986: 140; Pillai 1986: 153; Veron 1986: 320–321; Chevalier and Beauvais 1987: 710; Veron 1992: 123; Veron 1993: 199; Latypov 1995: 88; Nishihira and Veron 1995: 234; Veron 2000: 236; Suharsono 2004: 191; Claereboudt 2006: 187; Latypov 2006: 178; Suharsono 2008: 215; Wallace et al. 2009: 46.

*Diaseris* Milne Edwards and Haime 1849: 72; Milne Edwards and Haime 1850: xlix; Milne Edwards and Haime 1851: 117; Milne Edwards 1860: 54-55; Duncan 1883: 150; Gardiner 1905: 945; Veron and Pichon 1980: 119-121; Veron 1986: 326-327; Veron 1992: 127; Veron 1993: 205; Latypov 1995: 95; Nishihira and Veron 1995: 239; Veron 2000: 248; Suharsono 2004: 197; Claereboudt 2006: 190; Latypov 2006: 185; Suharsono 2008: 222. (Type species: *Fungia distorta* Michelin, 1842. Designation by monotypy.

*Fungia (Cycloseris)* – Hoeksema 1989: 30–31; Hoeksema and Van Ofwegen 2004.

##### Characters.

Adult corals either encrusting and polystomatous or free-living and monostomatous (Gittenberger et al. 2011, Benzoni et al. 2012). Outline of free-living, unfragmented specimens varying from circular to oval. Juveniles may be hexagonal. Free-living corals may fracture repeatedly into regenerating wedge-shaped pieces (Hoeksema 1989, Yamashiro et al. 1989, Yamashiro and Nishihira 1994, 1998, Hoeksema and Waheed 2011, 2012). Fragmenting corals may produce extra stomata along fracture lines. Corallum wall without perforations. Septal margins ornamented by fine, sharp dentations. Costae covered by fine spiny protuberances, which may become granular and blunt in large specimens. Tentacles small and usually translucent in extended state.

#### Cycloseris boschmai sp. n.

http://zoobank.org/8FA4CA99-7074-4425-A7ED-D051D6AB3311

http://species-id.net/wiki/Cycloseris_boschmai

Figures 3 –13

Fungia marginata (partim) Boschma 1923: 141–142; 1925: 199–202.
Fungia (Cycloseris) costulata (partim) – Hoeksema 1989: 64–69.
Fungia (Cycloseris) spec. – Hoeksema et al. 2004: 15; Hoeksema 2008: 11–12; 2010: 24–25.
Cycloseris sp. 1 – Gittenberger et al. 2011: 117; Hoeksema 2012a: 188.
Cycloseris sp. – Hoeksema et al. 2012: 652.
Cycloseris spec. – Waheed and Hoeksema 2013: 41.

##### Type material.

**Figure 3.**
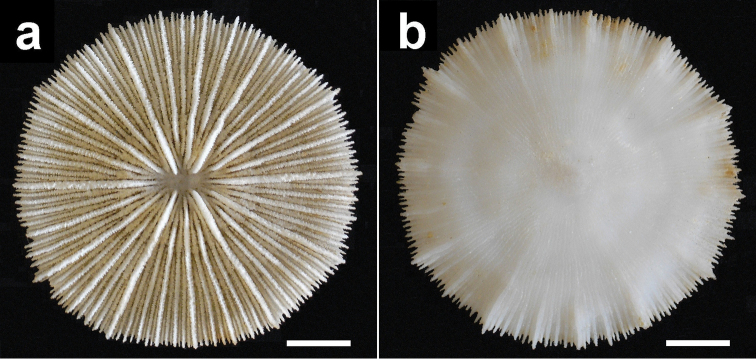
Holotype of *Cycloseris boschmai* sp. n. (RMNH Coel. 8333). Indonesia, Banda, Danish Exp. to the Kei Islands, 1922. **a** Upper side **b** Lower side. Scale bar: 0.5 cm.

Type specimens of *Cycloseris boschmai* from Banda, Moluccas, Indonesia (Danish Exp. to the Kei Islands, 1922), previously identified as *Fungia marginata* by Boschma 1925). **Holotype:** RMNH Coel. 8333 (1 dry specimen: 28 mm; Figure 3), Banda, 1922. **Paratypes:** RMNH Coel. 8334 (1 dry specimen: 29 mm), Banda, Lontor, 12.vi.1922; RMNH Coel. 8335 (5 dry specimens: 20-30 mm), Banda, off Lontor, 10-20 m depth, 4°33'S, 129°52'E, 1922.

##### Other material: Indonesia.

**Bali:** RMNH Coel. 40146 (2 dry specimens: 25, 31 mm), NE Bali, Tulamben, 3-5m, 08°16'36"S, 115°35'37"E, Bali Lombok Strait Exp. Sta. BAL.20, 09.iv.2001, coll. BWH; RMNH Coel. 40147 (1 dry specimen: 18 mm), NE Bali, Tulamben, 5m depth, 08°16'26"S, 115°35'28"E, Bali Lombok Strait Exp. Sta. BAL.21, 11.iv.2001, coll. BWH. **Nusa Tenggara Timur (Lesser Sunda Islands):** RMNH Coel. 21471 (1 dry specimen: 38 mm), NE Komodo, S Gili Lawa Laut, 08°27'00"S, 119°34'24"E, Snellius-II Exp. Sta. 4.253, 27.x.1984, coll. BWH; RMNH Coel. 40145 (4 dry specimens: 24–32 mm), SE Komodo, N side bay S of Tanjung Lohnamu, 08°38'19"S, 119°28'45’’ E, TNC Komodo Rapid Ecological Assessment Sta. KOM.16, coll. BWH; RMNH Coel. 31190 (1 dry specimens: 32 mm), N Sumbawa, Bay of Sanggar, 08°19'36"S, 118°15'12"E, Snellius-II Exp. Sta. 4.132, 30.x.1984, coll. BWH. **South Sulawesi.** RMNH Coel. 31188 (1 dry specimen: 31 mm), Spermonde Archipelago, N Bone Tambung Island, 05°01'50"S, 119°16'25"E, 13.vi.1986, coll. BWH; RMNH Coel. 31189 (5 dry specimens: 29–50 mm), Spermonde Archipelago, W Kudingareng Keke I., 9–18 m depth, 05°06'30"S, 119°17'04"E, 6.xii.1984, coll. BWH; RMNH Coel. 31192 (5 dry specimens: 17–31 mm), SW Selayer I., NW Bahuluang I., 06°28'00"S, 120°25'30"E, Snellius-II Exp. Sta. 4.202, 10.x.1984, coll. BWH; RMNH Coel. 31191 (3 dry specimens: 35–37 mm), NE Taka Bone Rate, E Tarupa Besar, 06°28'S, 121°08'E, Snellius-II Exp. Sta. 4.140, 25.ix.1984, coll. BWH. **Central Sulawesi, Tomini Bay, Togian Islands:** RMNH Coel. 24278 (2 dry specimens: 34, 39 mm), S Talatakoh I., 00°26'34"S, 122°06'07"E, Tethyana Exp. Sta. 10, 21.ix.1999, coll. BWH; RMNH Coel. 24291 (1 dry specimen: 48 mm), S Togian I., 00°20'10"S, 121°59'00"E, Tethyana Exp. Sta. 14, 23/24.ix.1999, coll. BWH; RMNH Coel. 24706 (5 dry specimens: 30-42 mm), S Batudaka I., 00°35'25"S, 121°41'38"E, Tethyana Exp. Sta. 15, 24.ix.1999, coll. BWH; RMNH Coel. 31193 (7 dry specimens: 29-50 mm), S Waleabahi I., 00°26'16"S, 122°15'16"E, Tethyana Exp. Sta. 8, 19.ix.1999, coll. BWH. **North Sulawesi:** RMNH Coel. 40156 (4 dry specimens; 36-40 mm), Lembeh Strait, Tanjung Mawali, 14 m depth, 01°26'36"N, 125°13'46"E, Lembeh Strait Exp. Sta. LEM.04, 31.i.2012, coll. BWH. **SE Sulawesi, Tukang Besi Islands (Wakatobi):** RMNH Coel. 40143 (1 dry specimen: 12 mm), NW Tomia, 05°43'59"S, 123°53'35"E, TNC-WWF Wakatobi Rapid Ecological Assessment Sta. WAK.25, 13.v.2003, coll. BWH; RMNH Coel. 40144 (1 dry specimen: 40 mm), SW Karang Kaledupa, lagoon, 05°51'46"S, 123°43'17"E, TNC-WWF Wakatobi Rapid Ecological Assessment Sta. WAK.28, 14.v.2003, coll. BWH. **Moluccas:** RMNH Coel. 33586 (2 dry specimens: 47, 58 mm), Ambon, N coast near Morela, 03°33'S, 128°12'E, Fauna Malesiana Maluku Exp. Sta. MAL.12, 13.xi.2002, coll. BWH; **Northern Moluccas:** RMNH Coel. 8286 (10 dry specimens, some with buds, 32-50 mm, previously identified as *Fungia marginata*), Banda, off Lontor, to 13 m depth, 4°33'S, 129°52'E, Danish Exp. to the Kei Islands, 15.vi.1922; UZMK (5 specimens in ethanol, 13-40 mm, previously identified as *Fungia marginata*), Gunung Api, 20-25 m depth, Danish Exp. to the Kei Islands,13.vi.1922; UZMK (9 specimens in ethanol, 26-50 mm, previously identified as *Fungia marginata*), Lontor, 13 m depth, Danish Exp. to the Kei Islands, 15.vi.1922; RMNH Coel. 40096 (1 dry specimen: 29 mm), Halmahera, East coast Teluk Dodinga, Karang Galiasa, 00°50'46"N, 127°35'07"E, Ternate Exp. Sta. TER.38, 14.xi.2009, coll. BWH; RMNH Coel. 40102 (1 dry specimen: 34 mm), Hiri I., Tanjung Ngafauda, 00°54'38"N, 127°19'03"E, Ternate Exp. Sta. TER.14, 16 m depth, 31.x.2009, coll. BWH; RMNH Coel. 40103 (1 dry specimen: 30 mm), Ternate, Sulamadaha I., 00°52'04"N, 127°19'33"E, Ternate Exp. Sta. TER.22, 18 m depth, 6.xi.2009, coll. BWH; RMNH Coel. 40104 (1 dry specimen: 19 mm), Ternate, Dufadufa, Benteng Toloko, 00°48'49"N, 127°23'22"E, Ternate Exp. Sta. TER.24, 8 m depth, 7.xi.2009, coll. BWH; RMNH Coel. 40105 (1 dry specimen: 31 mm), Halmahera, W Pasir Lamo, 00°53'21"N, 127°27'34"E, Ternate Exp. Sta. TER.26, 8.xi.2009, coll. BWH; RMNH Coel. 40106 (1 dry specimen: 48 mm), Tidore, north Pilongga, 00°42'50"N, 127°28'45"E, Ternate Exp. Sta. TER.34, 12 m depth, 12.xi.2009, coll. BWH; RMNH Coel. 40173 (1 dry specimen: 29 mm), Ternate, Tanjung Tabam, 00°50'05"N, 127°23'10"E, Ternate Exp. Sta. TER.12, 11 m depth, 30.x.2009, coll. BWH; RMNH Coel. 40174 (1 dry specimen: 17 mm), Ternate, outside harbor to the east, 00°46'55"N, 127°30'20"E, Ternate Exp. Sta. TER.25, 8 m depth, 7.xi.2009, coll. BWH. **East Kalimantan, Berau Islands:** RMNH Coel. 31922 (1 dry specimen: 19 mm), W Derawan I., 7 m depth, 02°16'53"N, 118°13'39"E, East Kalimantan Berau Exp. Sta. BER.02, 4.x.2003, coll. BWH; RMNH Coel. 31923 (1 dry specimen: 15 mm), Berau Delta, Lighthouse-2 Reef, 6 m depth, 02°09'34"N, 118°10'11"E, East Kalimantan Berau Exp. Sta. BER.05, 5.x.2003, coll. BWH; RMNH Coel. 31924 (2 dry specimen: 22, 31 mm), Samama I., 8 m depth, 02°07'32"N, 118°20'10"E, East Kalimantan Berau Exp. Sta. BER.10, 7.x.2003, coll. BWH; RMNH Coel. 31925 (1 dry specimen: 42 mm), NE Kakaban I., 14 m depth, 02°08'53’’ 118°32'32"E, East Kalimantan Berau Exp. Sta. BER.49, 28.x.2003, coll. BWH; RMNH Coel. 40149 (1 dry specimen: 14 mm), S of Samama I., NE Buliulin I., 14 m depth, 02°07'07"N, 118°20'32"E, East Kalimantan Berau Exp. Sta. BER.26, 15.x.2003, coll. BWH; RMNH Coel. 40153 (1 dry specimen: 46 mm), N Maratua I., near entrance lagoon, 9 m depth, 02°14'53"N, 118°37'36"E, East Kalimantan Berau Exp. Sta. BER.29, 17.x.2003, coll. BWH; RMNH Coel. 40154 (1 dry specimen: 21 mm), S Derawan I., 13 m, 02°15'04"N, 118°15'04"E, East Kalimantan Berau Exp. Sta. BER.04a, 18.x.2003, coll. BWH; RMNH Coel. 40155 (1 dry specimen: 50 mm), E Sangalaki I., Lighthouse, 12 m depth, 02°04'54"N, 118°24'30"E, East Kalimantan Berau Exp. Sta. BER.22, 14.x.2003, coll. BWH. **West Papua, Raja Ampat Islands:** RMNH Coel. 40140 (1 dry specimen: 30 mm), S. Mansuar (Sawandarik village), 00°35'26"S, 130°36'12"E, Raja Ampat Exp. Sta. RAJ.06, 20.xi.2007, coll. BWH; RMNH Coel. 40141 (1 dry specimen: 33 mm), Yeffam I., E Penemu I., 8 m depth, 00°35'20"S, 130°17'06"E, Raja Ampat Exp. Sta. RAJ.66, 13.xii.2007, coll. BWH. **Malaysia, Sabah, northern Borneo:** RMNH Coel. 33545 (1 dry specimen: 20 mm), W Sabah, Gaya Islands off Kota Kinabalu, W Sapi I., 06°00'26"N, 116°00'13"E, 28.vii.2005, coll. BWH. **Layang-Layang:** RMNH Coel. 40095 (2 dry specimens: 18 mm attached, 37 mm free-living), Easternmost point, Sta. LAC.14, 15-25 m depth, 07°22'34"N, 113°51'15"E, 28.iii.2013, coll. BWH. **Philippines:** RMNH Coel. 24908 (2 dry specimens: 20, 32 mm), Cebu Strait, West of Bohol, NW Cabilao I., 09°53'20"N, 123°45'53"E, 2.x.1999, coll. BWH. **Palau:** RMNH Coel. 40225 (1 dry specimen), SE off Garreru I., S Goraklbad Passage, 07°19'15"N, 134°35'50"E, 29.vii.2002, coll BWH.

##### Characters.

**Figure 4.**
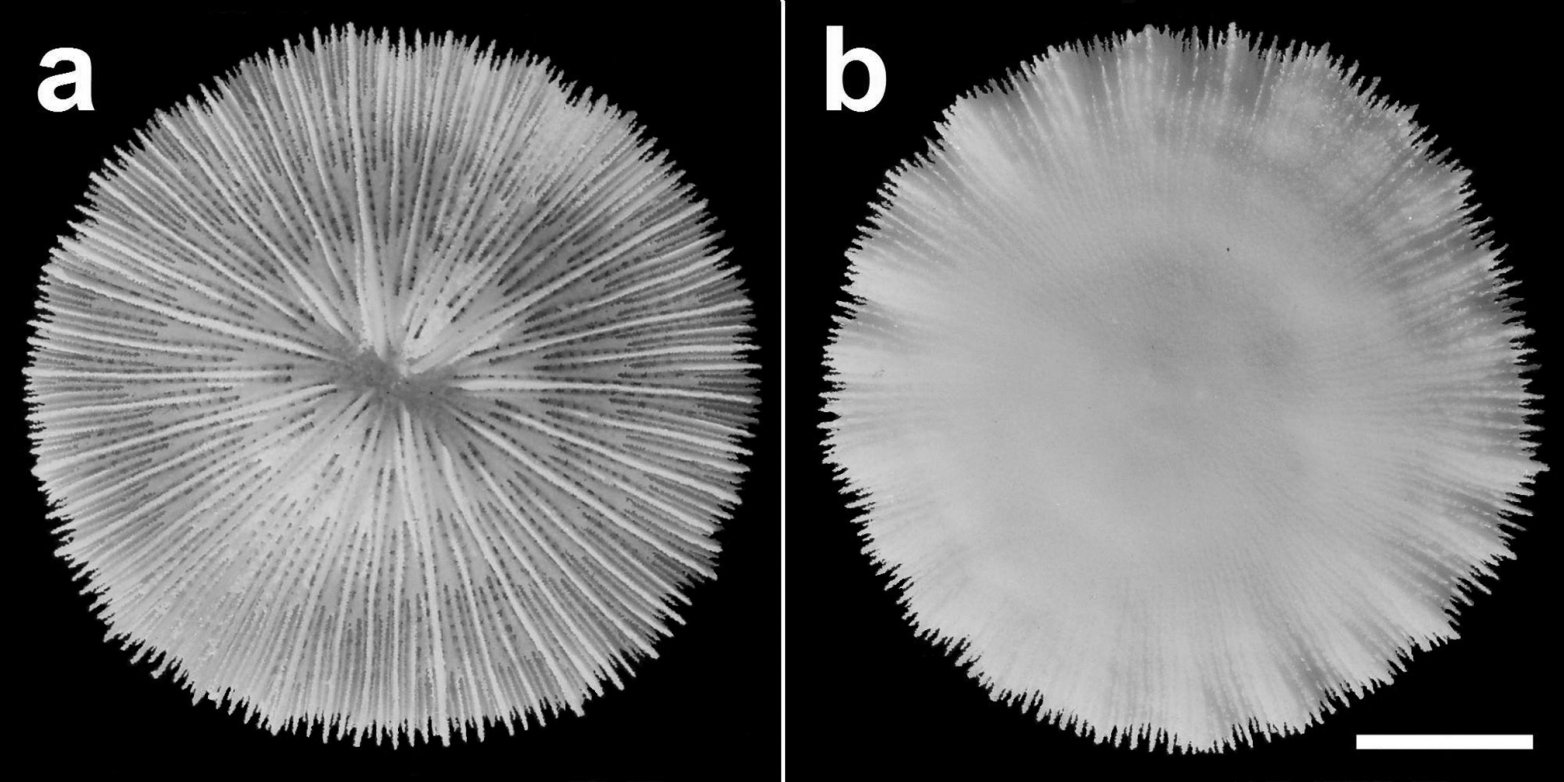
Specimen of *Cycloseris boschmai* sp. n. (RMNH Coel. 21471). Indonesia, NE Komodo, S Gili Lawa Laut, Snellius-II Exp. Sta. 4.253, 27 October 1984. **a** Upper side **b** Lower side. Scale bar: 0.5 cm.

**Figure 5.**
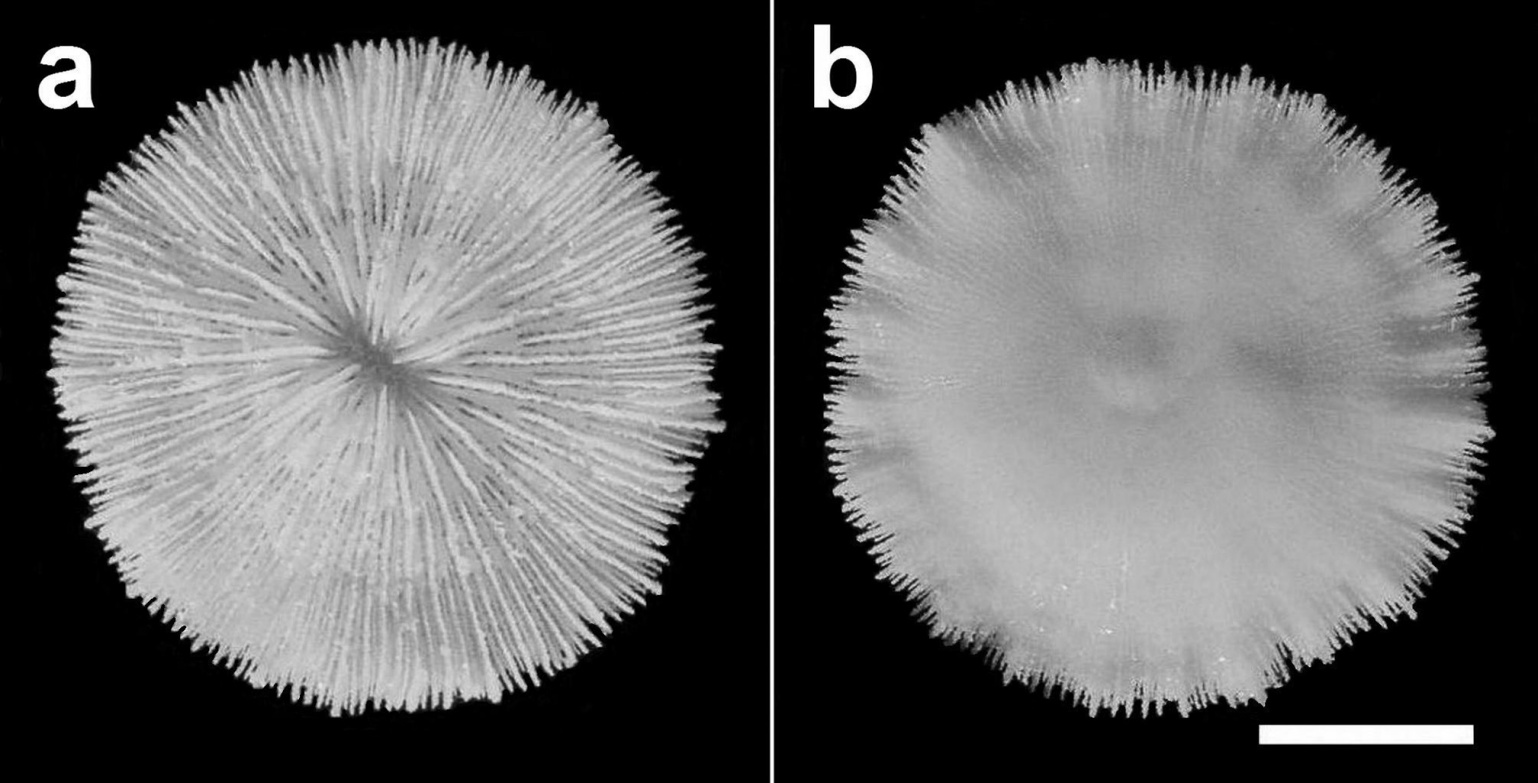
Specimen of *Cycloseris boschmai* sp. n. (RMNH Coel. 31190). Indonesia, N Sumbawa, Bay of Sanggar, Snellius-II Exp. Sta. 4.132, 30 October 1984 **a** Upper side **b** Lower side. Scale bar: 0.5 cm.

**Figure 6.**
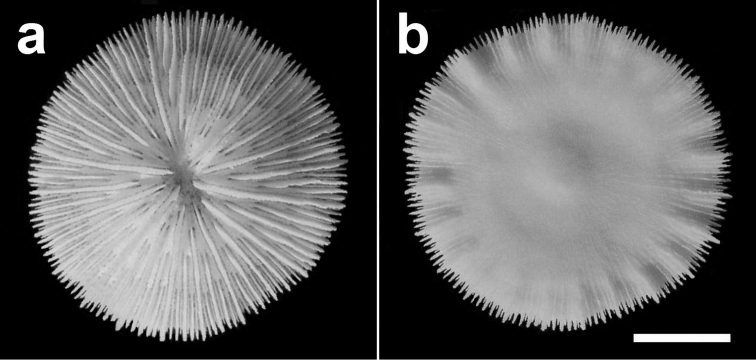
Specimen of *Cycloseris boschmai* sp. n. (RMNH Coel. 31188). Indonesia, South Sulawesi, Spermonde Archipelago, north side of Bone Tambung Island, 13 June 1986. **a** Upper side **b** Lower side. Scale bar: 0.5 cm.

**Figure 7.**
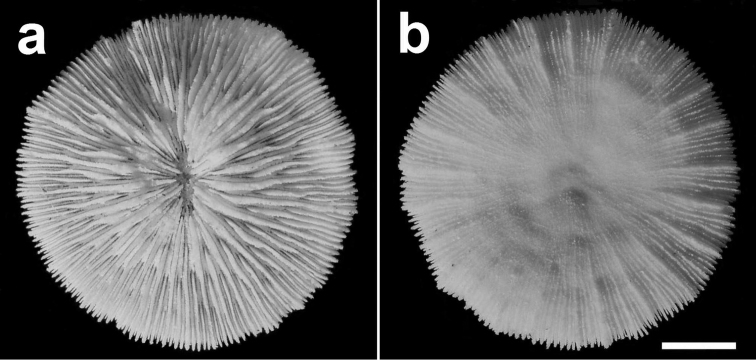
Specimen of *Cycloseris boschmai* sp. n. (RMNH Coel. 24291). Indonesia, Central Sulawesi, Togian Islands, S Togian I., Tethyana Exp. Sta. 14, 23/24 September 1999 **a** Upper side **b** Lower side. Scale bar: 0.5 cm.

**Figure 8.**
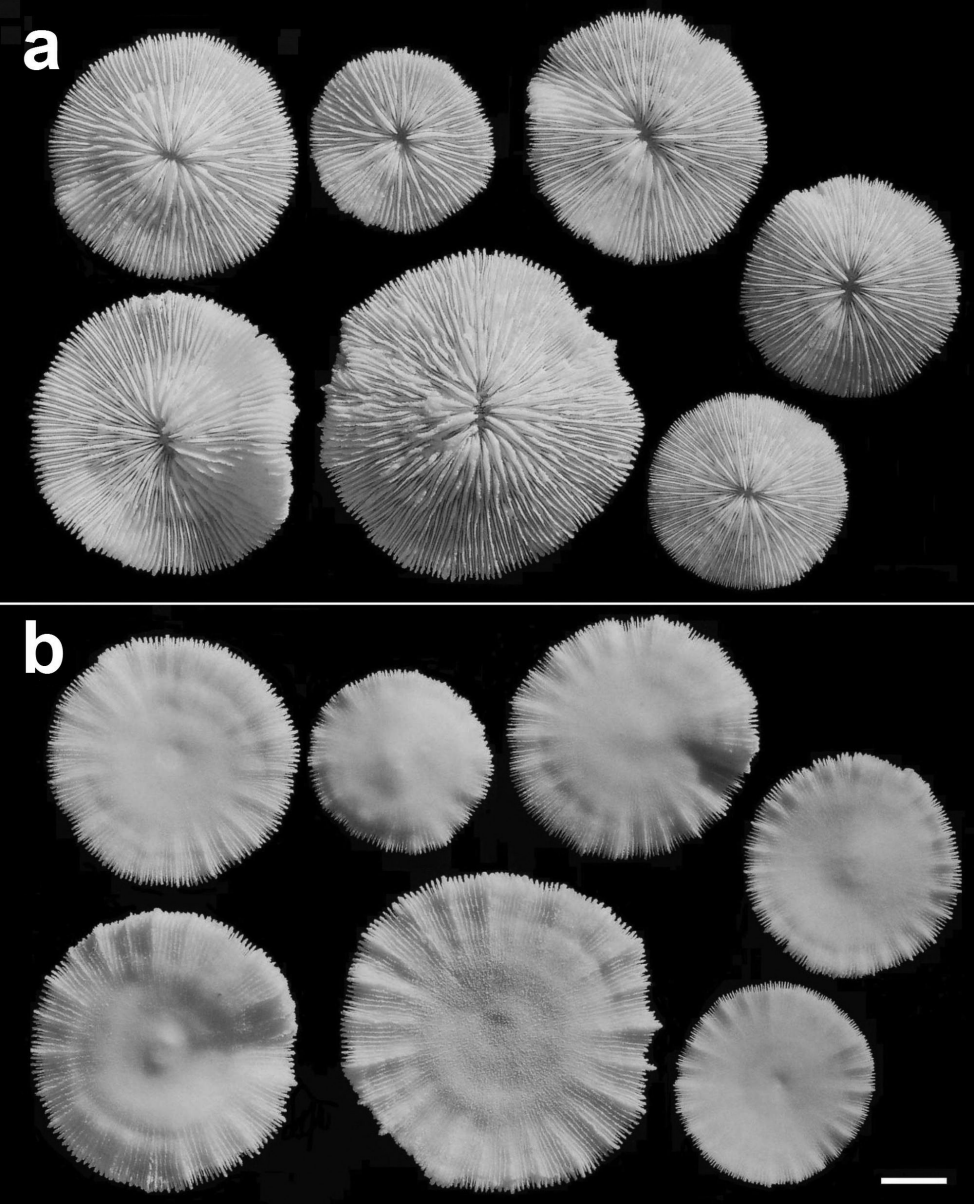
Specimens of *Cycloseris boschmai* sp. n. (RMNH Coel. 31193). Indonesia, Central Sulawesi, Togian Islands, S Waleabahi I., Tethyana Exp. Sta. 8, 19 September 1999 **a** Upper side **b** Lower side. Scale bar: 0.5 cm.

**Figure 9.**
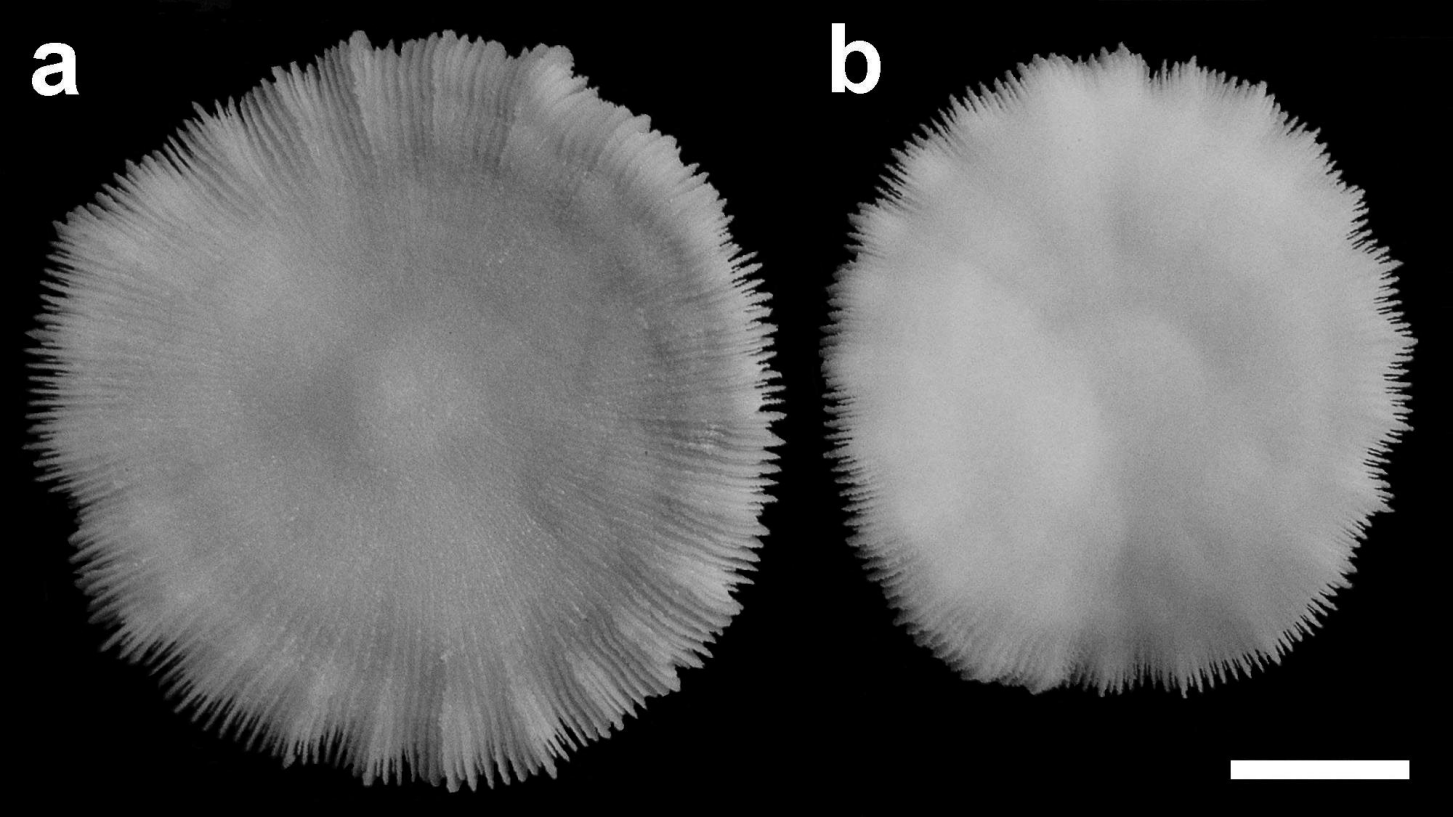
Two specimens of *Cycloseris boschmai* sp. n. (RMNH Coel. 24278). Indonesia, Central Sulawesi, Togian Islands, S Talatakoh I., Tethyana Exp. Sta. 10, 21 September 1999. Scale bar: 0.5 cm.

**Figure 10.**
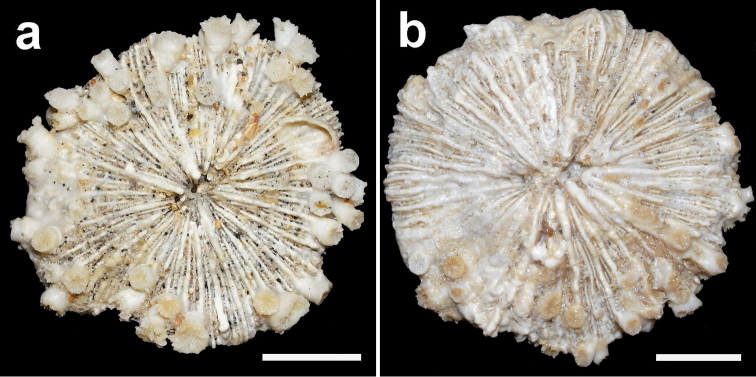
Two specimens of *Cycloseris boschmai* sp. n. (RMNH Coel. 8286) with marginal buds and sand in the mouths. Indonesia, Banda, off Lontor, Danish Exp. to the Kei Islands, 15 June 1922.

**Figure 11.**
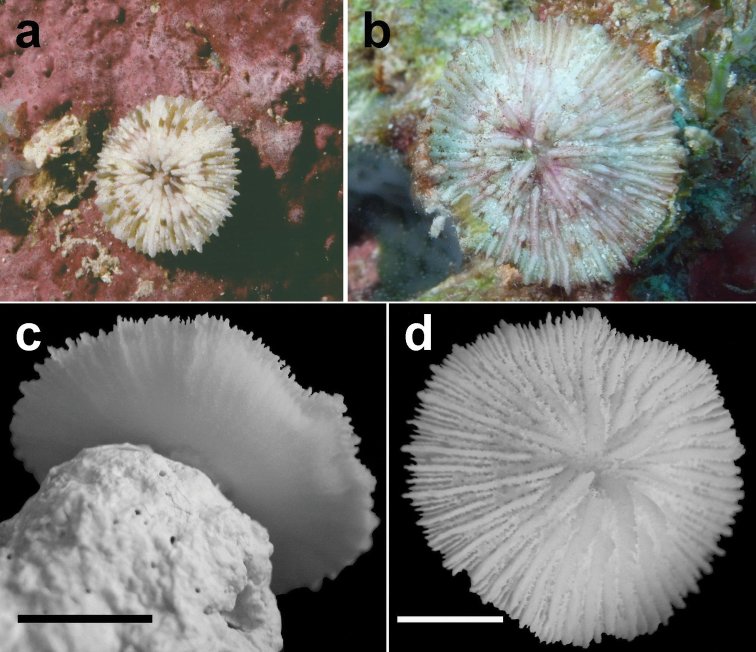
Juvenile, attached specimens of *Cycloseris boschmai* sp. n. **a** Papua New Guinea, Bismarck Sea, Madang, June 1992 **b–d** Malaysia, South China Sea, Layang Layang, Easternmost point, (RMNH Coel. 40095), 28 March 2013 **b** In situ (bleached) **c** Lower side **d** Upper side. Scale bars: 0.5 cm.

**Figure 12.**
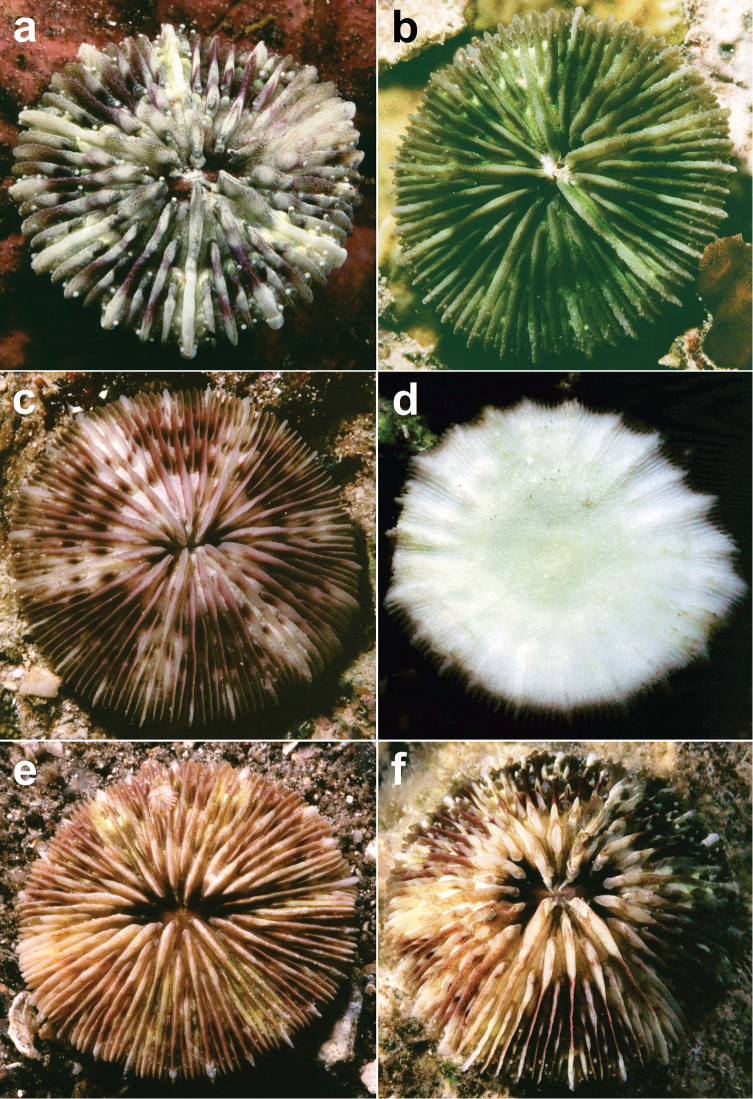
*Cycloseris boschmai* sp. n. **a** Indonesia, Bali, Tulamben, September 1997 **b** Philippines, Cebu, November 1999 **c–e** Indonesia, Central Sulawesi, Togian Islands, September 1999 **f** Indonesia, South Sulawesi, Spermonde Archipelago, Bone Lola reef, August 1997.

**Figure 13.**
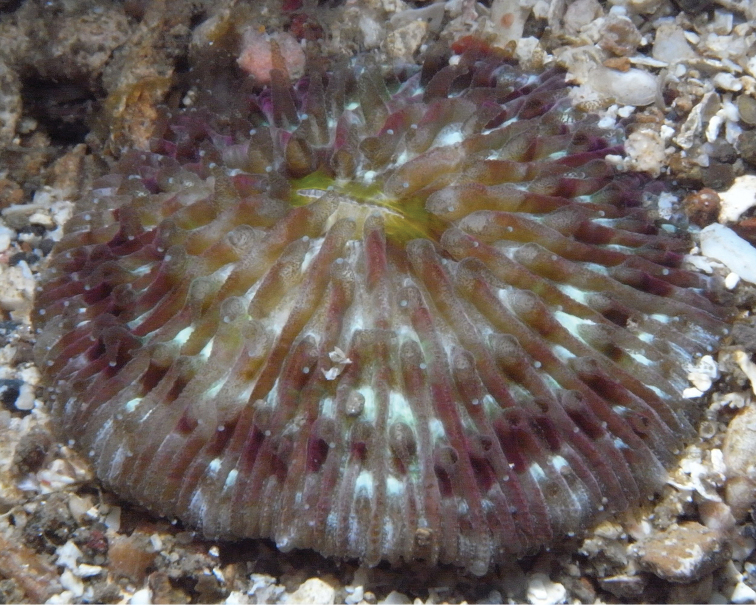
*Cycloseris boschmai* sp. n. specimen showing transparent extended tentacles with white acrospheres at their tips; Indonesia, North Sulawesi, Lembeh Strait, Lobangbatu, February 2012.

(Figures 3–13) The diameter of the examined specimens ranges between 12 and 50 mm. Corals mostly flat, moderately thick and robust. Adult animals unattached and monostomatous with septa-costae extending outside the circular to slightly oval corallum outline. Juvenile specimens vary from round to slightly hexagonal. Wedge-shaped, regenerating fragments not known. The length of the fossa, measured at its bottom, is 1/9 to 1/6 of the corallum length. The columella is formed by a mingled mass of tightly to loosely packed trabeculae. Septa densely packed and straight, unequal in thickness and height. The relatively thick and high septa of lower orders are solid; they are flanked by perforated septa of higher orders. Tentacular lobes absent. Septal margins are finely ornamented with sharp and granular dentations. Their number varies from 20 to 70 per cm. Septal sides are densely covered by fine granulations, which are irregularly dispersed or arranged in rows perpendicular to the septal margin. Compound synapticulae (fulturae) connecting the septa laterally cannot easily be distinguished because of tight septal arrangement. The solid corallum wall is granulated and may show a detachment scar. The lower side varies from flat to slightly convex. Costae unequal in size, straight and prominent near the corallum margin but less distinct at the centre. Corallum margin may be slightly undulating because of enlarged lower order costae. Costae ornamented with fine granular or acute spines. Their number varies from 15 to 80 per cm. Some individuals have small buds over their surface, especially in the proximity of the corallum margin (Figure 10). Attached juveniles (anthocaulus stage) are rare (Figure 11). The color of the living animal is variable with hues of red or green (Figures 11–13). Tentacles small and transparent with white acrospheres at their tips (Figure 13).

##### Geographical distribution

**Figure 14.**
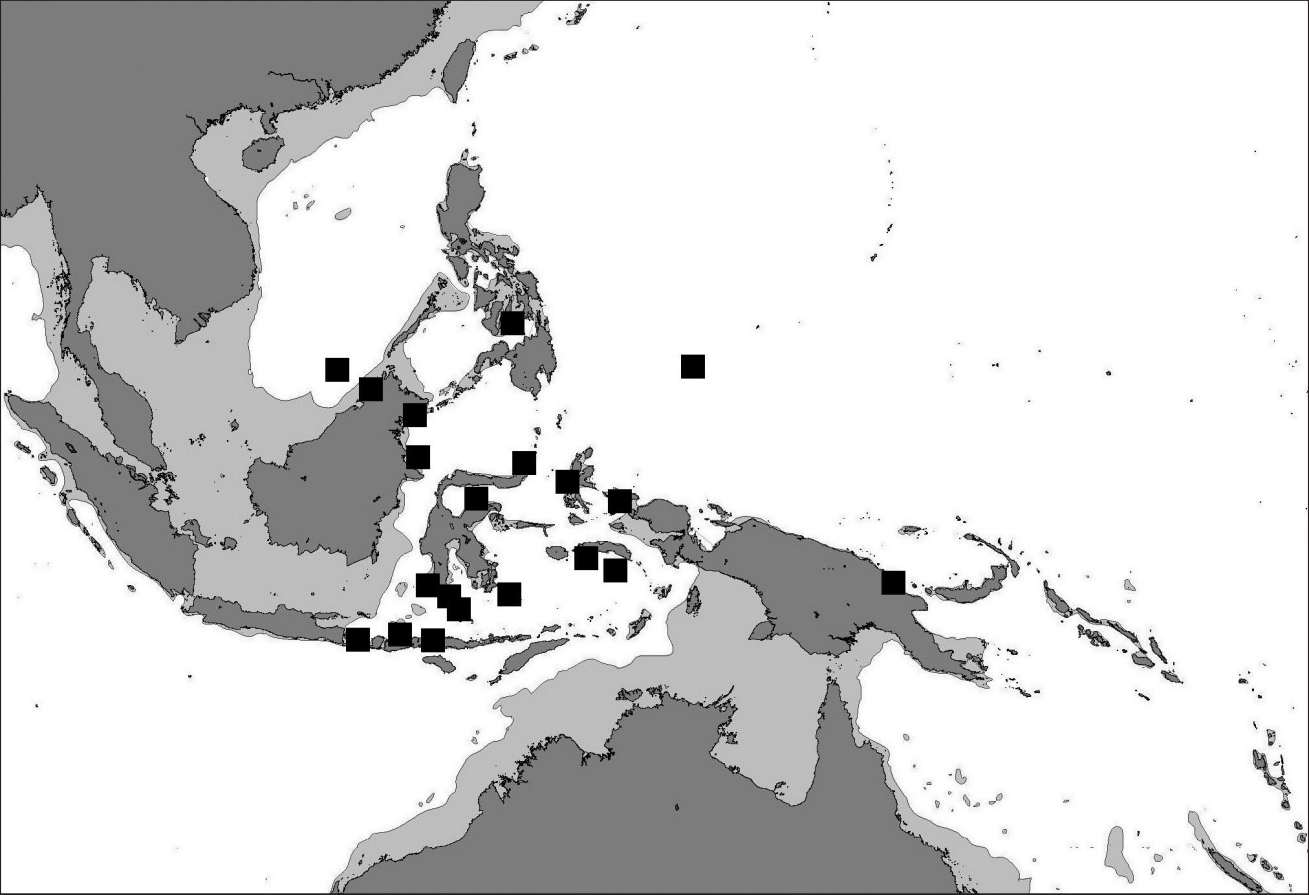
Map of the Central Indo-Pacific indicating localities where *Cycloseris boschmai* sp. n. has been recorded.

(Figure 14). The distribution range is limited to the Coral Triangle (Hoeksema 2007): eastern Malaysia (Sabah and Layang Layang), eastern Indonesia (from Bali to West Papua), central Philippines (Cebu Strait), Papua New Guinea (Madang Lagoon), and Palau.

##### Etymology.

The species is named after the late Prof. Hilbrand Boschma, former director of the Rijksmuseum van Natuurlijke Historie (now Naturalis Biodiversity Center), who devoted much of his research time to the study of mushroom corals, including specimens of the new species.

##### Diagnosis.

Adult corals small (< 50 mm) with uneven circular corallum margin owing to enlarged costae. Live specimens with variable, patchy colouration.

#### Key to recent free-living *Cycloseris* species (full-grown, unfragmented specimens), partly after Hoeksema (1989)

**Table:** 

1a	Lower order costae distinctly larger than other ones	2
1b	Costae fine, adjacent ones equal to almost equal	5
2a	Coralla flat and thin, corallum outline circular	3
2b	Coralla thick and slightly arched, corallum outline slightly or much oval	4
3a	Lower order costae thicker and longer than higher order costae, ornamentation fine (20–70 / cm), maximum corallum diameter 5 cm, habitat mostly consisting of reef slopes and sandy reef bases	*Cycloseris boschmai* sp. n.
3b	Lower order costae sharp, ornamentation very fine (40–80 / cm) on lower order costae and indistinct on higher order costae, maximum corallum diameter 8 cm, habitat mostly consisting of deep, sandy reef bases	*Cycloseris vaughani*
4a	Corallum outline slightly oval, lower order costae irregularly and roughly ornamented (20–70 / cm), maximum corallum diameter 8.5 cm, habitat consisting of upper reef slopes	*Cycloseris tenuis*
4b	Corallum outline clearly oval, lower order costae sharp, costal ornamentation very fine (40–90 / cm) and nearly absent on higher order costae, maximum corallum diameter 12.5 cm, habitat mostly consisting of deep, sandy reef bases	*Cycloseris somervillei*
5a	Septa densely packed and (almost) equal in height	6
5b	Septa loosely packed, septa of lower orders thicker and more exsert than others	7
6a	Central fossa short (< 10% of corallum diameter); all septa perforated, nearly equal in size and tightly packed with little space in between them, maximum corallum diameter 8.5 cm, habitat mostly consisting of deep, sandy reef bases	*Cycloseris sinensis*
6b	Length of central fossa > 10% of corallum diameter, septa of lower order solid and thicker than adjacent septa with distinct space in between them, maximum corallum diameter 7.5 cm, habitat consisting of deeper reef slopes or reef bases	*Cycloseris distorta*
7a	Corallum outline oval	8
7b	Corallum outline circular or irregularly round with folds or undulations	9
8a	Coralla thick; underside flat or arched, costae equal, maximum corallum diameter 9 cm, habitat consisting of lower reef slopes or sandy reef bases	*Cycloseris cyclolites*
8b	Coralla convex around fossa (humped), costae equal in juveniles, maximum corallum diameter 12.5 cm, habitat mostly consisting of deep, sandy reef bases	*Cycloseris somervillei*
9a	Coralla with folded, undulating margin	10
9b	Coralla with regular, smooth periphery	11
10a	Coralla thin, central fossa short (< 10% of corallum diameter), margin undulating (hexagonal in juveniles) maximum corallum diameter 8.5 cm, habitat consisting of sandy reef slopes or sandy reef bases	*Cycloseris hexagonalis*
10b	Coralla thick and usually strongly arched, margin with folds, maximum corallum diameter 8.5 cm, habitat consisting of lower reef slopes or sandy reef bases	*Cycloseris curvata*
11a	Coralla and septa thin, adjacent costae slightly alternating in size maximum corallum diameter 15 cm, habitat mostly consisting of sandy reef bases	*Cycloseris fragilis*
11b	Coralla moderately thick, lower order septa thicker than others, costae nearly similar in size, maximum corallum diameter 12 cm, habitat consisting of lower reef slopes or sandy reef bases	*Cycloseris costulata*

## Discussion

Although some material of *Cycloseris boschmai* sp. n. was already available in museum collections (RMNH, UZMK), the species could only be discovered because of much fieldwork (1983–2013) with proportionate opportunities for observations and sampling to enable separation of the new species from resembling ones. Boschma (1923a, 1925) might have had the same species in mind when he described and studied *Fungia marginata*, but his selection of type specimens from the Paternoster Islands and the unclear comparison with other species of Döderlein's, *Fungia patella* group are not convincing. He described this species because of its supposedly thick corallum margin as compared to the other species in the *Fungia patella* group, but this character is not useful when applied several other *Cycloseris* species.

The *Fungia marginata* material from Banda is suitable as type material of the new species. Because the Banda specimens were wrongly identified by him, this does not concern a new name for an existing species but an entirely new species (Hoeksema 1993b). These old museum specimens are not just useful as type material but also because they supply information about habitat (field data) and asexual reproduction by budding. They also constitute the oldest material of *Cycloseris boschmai*, which gives them potential historical value as baseline material in studies on changing coral faunas (Hoeksema and Koh 2009, Van der Meij et al. 2010, Hoeksema et al. 2011, Van der Meij and Visser 2011, Hoeksema and Wirtz 2013).

*Cycloseris boschmai* sp. n. is the smallest mushroom coral known so far (see key). Superficially, it resembles *Cycloseris costulata*, which has less prominent costae, a more even corallum margin (not undulating), a larger maximum size and less colourful appearance (see Hoeksema 1989, Hoeksema and Van Ofwegen 2004, Gittenberger and Hoeksema 2006). Both species can be found on reef slopes and sandy reef bases. *Cycloseris costulata* is common and wide-spread (Hoeksema 1989, 2012a) and has a variable growth form (Hoeksema and Moka 1989, Gittenberger and Hoeksema 2006), which is why much material had to be examined to find sufficient consistency in the diagnostic characters of the new species.

*Cycloseris boschmai* sp. n. also resembles *Cycloseris tenuis*, which is more oval, less corlourful (see Gittenberger and Hoeksema 2006), and with lower order costae that are rougher and more irregularly ornamented. *Cycloseris tenuis* is most common on upper reef slopes (Hoeksema 2012a), whereas the rarer *Cycloseris boschmai* shows a deeper depth range. The new species also resembles *Cycloseris vaughani*, which has sharper lower order costae, a brown colouration (Hoeksema 1989, Hoeksema and Van Ofwegen 2004) and a much deeper depth range (Hoeksema 2012a).

With the inclusion of *Cycloseris boschmai* sp. n., 11 free-living *Cycloseris* species are distinguished. Since the taxonomic revision of the Fungiidae by Hoeksema (1989) various other mushroom coral species were reported as new to science (Veron 1990, 2000, 2002, Hoeksema and Dai 1991, Hoeksema 1993a, 1993c, 2009, 2012c, Latypov 1995, 2006, Ditlev 2003, Mondal and Raghunathan 2013). Two of these were originally classified as *Cycloseris* but they appear to be synonyms of previously described species and one of these is not a *Cycloseris*.

*Cycloseris colini* Veron, 2000 is a synonym of *Lithophyllon spinifer* (Claereboudt and Hoeksema 1987). The central dome and upward margins, combined with the large corallum size as described by Veron (2002) are characters commonly found in *Lithophyllon spinifer* (see Hoeksema 1993a: fig. 14, Veron 2000, Hoeksema and Van Ofwegen 2004, Hoeksema 2008: fig. 9). A specimen from Palau (MTQ G55817) was designated holotype by Veron in 2002, but since the species was described in 2000 this designation was invalid (ICZN 2011). Hence, this specimen is hereby designated lectotype.

*Cycloseris densicolummelus* Latypov, 2006 has not been described in an official publication but in an electronic document that was distributed via a CD-ROM. This work should have contained a clear publication date and a statement naming at least five major publicly accessible libraries in which copies of the optical disc were to have been deposited (ICZN 2012). Since only the year of translation has been mentioned in the introduction and no names of libraries were given, this name is not valid. Although the publication by Latypov (2006) is said to be an English translation of an original book in Russian, the latter does not mention *Cycloseris densicolummelus* but “*Cycloseris* sp. 1” (Latypov 1995: 95, pl. 28 fig. 2). The specimen indicated as “holotype”, spec. 1/95158 deposited in the Museum of Institute Marine Biology, Vladivostok 69041, Russia, is from Mai Rut Island, Gulf of Thailand. The illustration with the species description (Latypov 2006: figs 46–8) shows that it is a specimen of *Cycloseris costulata*. The well developed tentacular lobes, the intensive granulation of the lateral septal surfaces and the densely packed trabeculae of columella, which are indicated as diagnostic characters, do not really distinguish *Cycloseris densicolummelus* from *Cycloseris costulata* (see Hoeksema 1989, Gittenberger and Hoeksema 2006).

Because *Cycloseris boschmai* is a rare species (considering that most material was gathered during fieldwork in a time span of 30 years) and its geographic distribution range is restricted to the Coral Triangle, not much can be said about its ecology. Specimens are difficult to find, owing to their small body size compared to other mushroom coral species (Hoeksema 1991b, Gittenberger et al. 2011), which may be restrictive to the settlement of associated fauna and therefore none of its symbionts was reported previously (Hoeksema et al. 2012: “*Cycloseris* sp.”). In the present study, one of the photographed specimens shows a coral barnacle (Figure 11e), which is now the only known associated animal.

Small-sized free-living mushroom corals have been reported to show much mobility (Hoeksema 1988, Yamashiro and Nishihira 1995), which may help them to escape from competition for space with other organisms (Chadwick 1988, Hoeksema and De Voogd 2012). The distinctive large lower order costae may be useful as ridges in stabilizing the corals in order to prevent them to slide too rapidly downslope to deeper reef zones with sandy substrate (Hoeksema 1988). The enlarged ridge-like costae can also be seen in some other *Cycloseris* species (see key) and in the small-sized free-living deep-sea coral *Deltocyathus rotulus* (Alcock, 1898) (see Cairns and Kitahara 2012: fig 18Q).

Although free-living *Cycloseris* species were previously considered primitive, this is not the case according to their phylogeny reconstruction (Gittenberger et al. 2011). Their predominant habitat of deeper sandy substrates can also be considered an advanced trait (Hoeksema 2012b). The sand in the mouth of nearly dead specimens from Banda (Figure 10) suggests that they were collected from a sandy substrate, which may not be their preferred habitat. During the author's, fieldwork (1983–2013), no specimens were observed on sandy reef bases. *Cycloseris boschmai* corals are small but not thin, which may not facilitate mobility and sediment-shedding as seen in large-polyped corals (Bongaerts et al. 2012, Erftemeijer et al. 2012). The presence of buds in several of the specimens from Banda, in addition to the sand in their stomata, (Boschma 1925; Figure 10) may also indicate that specimens have been buried (Gilmour 2002). Specimens of *Cycloseris hexagonalis* have been observed to show a similar abundance of buds on a sandy slope in eastern Sabah (BWH personal observation 2009). Budding may be a mushroom coral's, last resort of survival when its mouth is clogged and not capable of food intake anymore (Boschma 1922, 1923b, 1923c).

## Supplementary Material

XML Treatment for Cycloseris

XML Treatment for Cycloseris boschmai
